# Effects of physical activity interventions in frail and prefrail community-dwelling people on frailty status, muscle strength, physical performance and muscle mass—a narrative review

**DOI:** 10.1007/s00508-019-1484-7

**Published:** 2019-04-02

**Authors:** Sandra Haider, Igor Grabovac, Thomas E. Dorner

**Affiliations:** Department of Social and Preventive Medicine, Centre for Public Health, Medical University of Vienna, 1090 Vienna, Austria

**Keywords:** Exercise training, Intervention studies, Frailty, Older adults

## Abstract

**Background:**

Frailty is a geriatric syndrome, which is highly prevalent in community-dwelling older adults and is associated with a variety of unwanted health outcomes, including dependency and institutionalization. Physical activity (PA) interventions may be of great importance in frail people to improve the frailty status, muscle strength, physical performance and muscle mass.

**Methods:**

A narrative review of randomized-controlled trails was performed, including frail and prefrail community-dwelling older adults. Included were studies with different PA interventions, such as aerobic activity, strength and balance training, stretching, and a combination of these methods.

**Results:**

Overall, 14 studies were included. The PA interventions led to a significant reduction in the frailty status (3/5 studies), to an increase in muscle strength (4/8 studies), to improved physical performance (7/11 studies), and to an increase in muscle mass (1/4 studies), when compared to the control group. The studies analyzed differed in various aspects of study protocols (training protocol, intensity, frequency, follow-up time, measuring tools) and delivery method of intervention (health professionals, lay volunteers, at home in health care institutions).

**Conclusions:**

Although it was not consistently reported in the studies that PA interventions are successful in increasing muscle mass in frail and prefrail older people, the results support the effectiveness of PA interventions on the reduction of frailty, and the increase in muscle strength and physical performance.

## Introduction

**Fig. 1:**
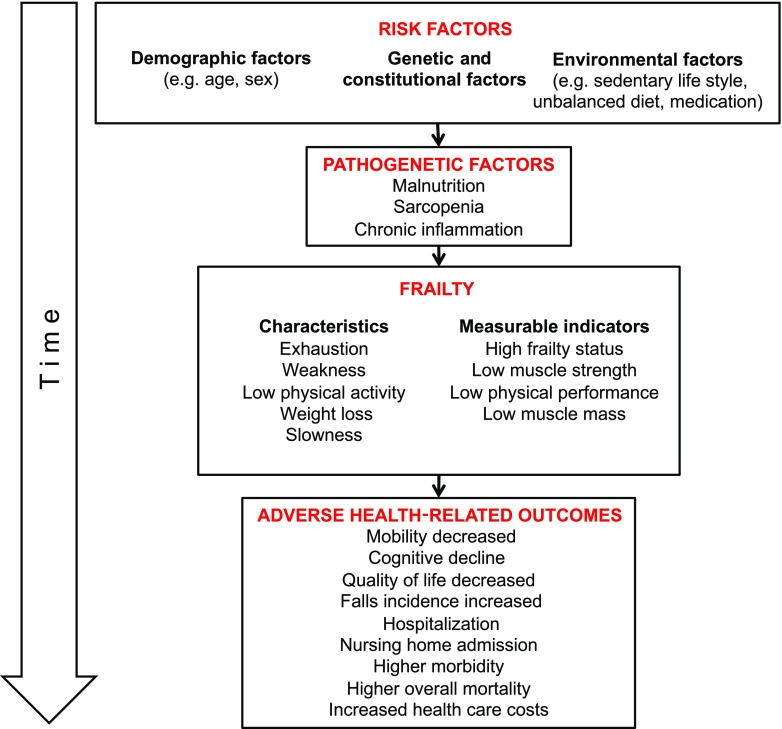
Flow diagram showing pathway of frailty—From risk factors to the consequences

With the demographic shift and the rising of population age in most continental world regions [1], the health burden of aging-related morbidities presents as a major public health issue [2]. In order to conceptualize the influence of a variety of factors associated with aging-related outcomes, the concept of frailty has been proposed [3]. Although a standardized definition has not yet been established [4], frailty as a geriatric syndrome has been characterized by increased vulnerability to external stressors as a result of malnutrition, sarcopenia and chronic inflammation [5]. The most commonly accepted concept of frailty is the frailty phenotype of the Cardiovascular Health Study (CHS), in which frailty is defined as the presence of minimum 3–5 different criteria; unintentional weight loss, self-reported exhaustion, weakness, slow walking speed and low physical activity [5]. Although the pathway and the pathophysiological development of frailty is not known, this geriatric syndrome has been named as a predictor of numerous adverse health-related outcomes including mobility decrease and cognitive decline, lower quality of life, high frequency of falls, hospital and nursing home admissions, various morbidities as well as overall mortality ([5–7]; Fig. 1). Furthermore, frailty status, muscle strength, physical performance, and muscle mass are among the measurable indicators of frailty (Fig. 1).

**Fig. 2:**
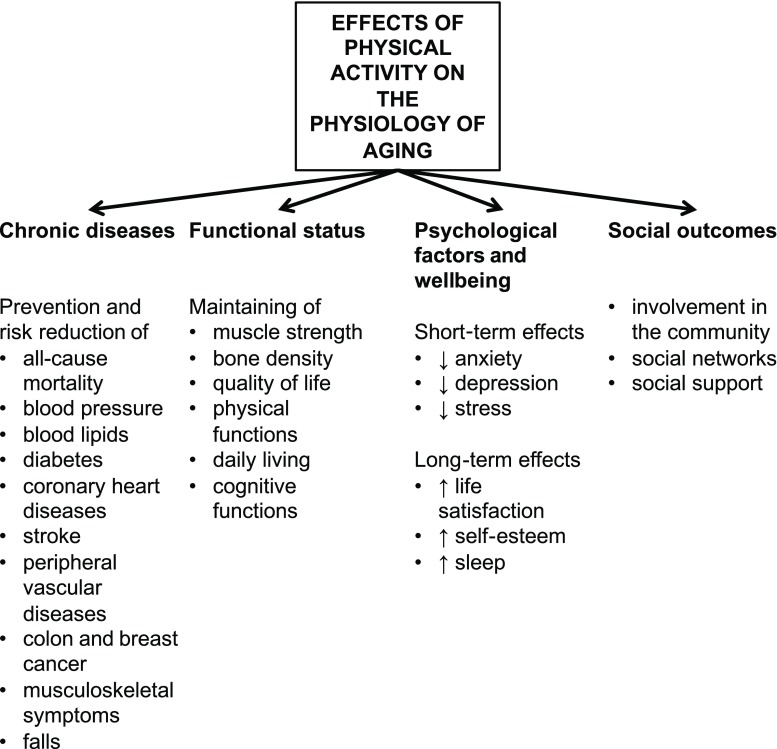
Effects of physical activity on aging

The overall prevalence of frailty differs between countries and settings (community-dwelling vs. long-term care units). In community-dwelling people, a 2004 analysis of the Survey of Health, Aging and Retirement in Europe (SHARE) data from 10 European countries indicated an overall prevalence of frailty at 17% in people ≥65 years of age [2]. Additionally, 42% were labelled as prefrail.

Based on the literature, physical activity (PA) interventions might be an important tool to counteract frailty [8, 9]. Here PA is defined as any bodily movement produced by skeletal muscles, with the subdomains occupational, sports, conditioning, household, and other activities [10]. In older adults, regular PA is associated with a large number of potential health benefits (Fig. 2). These include prevention and reduction of numerous chronic illnesses, improvement in functional as well as psychosocial status [11]. Nonetheless, PA is a major public health problem and according to the Eurostat, including 28 countries of the European Union, only 22.9% of older adults fulfil the minimum criteria for aerobic physical activity [12, 13]. Based on the recommendations, multicomponent PA regimes (comprising balance, strength and endurance training) are recommended [14–16]. As frailty is associated with sarcopenia, strength training may be of most importance, as it increases muscle mass, strength and power [14]. Studies have shown that strength training has positive effects on older adults in terms of improving muscle strength, physical performance and muscle mass [11, 14, 17]; however, it is not clear if these effects also apply for people suffering from frailty. To our knowledge there are two reviews addressing the effects of exercise interventions on frailty, albeit these were not exclusively focused on community-dwelling older adults [18, 19].

With the apparent high prevalence of frailty and its associations with negative health outcomes, as well as the demographic changes, preventive measures to reduce the prevalence of frailty are of great public health importance. Given the growing number of publications dealing with effects of PA on frailty in community-dwelling older adults, often with conflicting results, this study aimed for the first time to summarize the current evidence for the effect of PA on four measurable frailty indicators: frailty status, muscle strength, physical performance, and muscle mass, in a narrative review.

## Methods

A narrative review was conducted to provide an overview of the topic while not saturating the literature. A Medline and Google Scholar search was conducted. The articles were screened and only randomized controlled trials were included that dealt with any kind of PA interventions (e. g. aerobic activity, strength and balance training, stretching, and a combination of these methods) in frail or prefrail community-dwelling older adults. Studies were only included if a validated operational definition of frailty (e. g. Fried criteria [5], Edmonton criteria [20] and SHARE Frailty Index [21]) was used. This was especially important given the reported differences in results between studies with validated operational vs. non-operational and non-validated definitions of frailty, where studies with non-operational and non-validated definitions reported higher effects [19]. It was of interest to see if PA reduced frailty, improved muscle strength and increased physical performance and if overall muscle mass was improved, and focused on 4 questions: (1) does PA reduce frailty in community-dwelling prefrail and frail persons? (2) Does PA improve muscle strength in community-dwelling prefrail and frail persons? (3) Does PA improve physical performance in community-dwelling prefrail and frail persons? (4) Does PA increase muscle mass in community-dwelling prefrail and frail persons?

## Results

Overall 14 studies were included and the results are presented in Tables 1, 2, 3 and 4.

**Table 1:** Overview of the effects of physical activity intervention on frailty status

Author, year	Sample	Intervention	Main findings
Cameron et al. 2013 [22]	216 frail adults ≥70 years	12 months *Intervention (I)* : multifactorial, individually tailored treatment 1) If weight loss criterion was met, a dietician came. 2) If exhaustion criterion was met and the Geriatric Depression Scale score was high, a psychiatrist or psychologist came. 3) If weakness, slowness or low energy expenditure criteria was present, 10 home-based physiotherapy sessions were performed *Control (C)*: usual care, including health and aged care services	Change of frail people in % acc. to the CHS criteria (Fried et al.) [5]. *I*: Baseline: 64%; 3 months: 64% 12 months: 62% *C*: Baseline: 65%; 3 months: 75%; 12 months: 76% → sig. group difference was 14.7% at 12 months Change in frailty status (Number of frailty criteria present) *I*: Baseline: 3.4 (SD: 0.7); 3 months: 0.6 (SD: 1.1); 12 months: 0.8 (SD: 1.2) *C*: Baseline: 3.5 (SD: 0.7); 3 months: 0.4 (SD: 0.9); 12 months: 0.4 (SD: 1.0) → sig. group difference
Chan et al. 2012 [23]	117 prefrail or frail adults; 65–79 years	3 months *Exercise and nutrition (EN)*: 3 × a week (1 h), structured exercise course warm up (15 min), brisk walks (10 min), stretching, strength training (10–15 rep., rubber band and bottled water), balance training, cool down (5 min) *Control (Non-EN)*: no intervention	Improvement of the frailty status by one category acc. to the CHS criteria (Fried et al.) [5]. *EN*: 3 months: 45%; 3 months: 42%; 12 months: 40% *Non-EN*: 3 months: 27%; 6 months: 26%; 12 months: 31% → sig. difference at 3‑month between EN and non EN
Luger et al. 2016 [21]	80 prefrail or frail persons; ≥65 years	3 months *Physical training and nutritional (PTN)*: 2 × a week; home visits by trained lay volunteers strength exercises (6 exercises, 2 sets, 15 rep until muscular exhaustion) and nutritional support *Social support (SoSu)*: 2 × a week; home visits with social support	Decrease in frailty prevalence—SHARE-FI (score) (Romero et al.) [37]. *PTN*: −17% *SoSu*: −16% → sig. decrease in both groups
Li et al. 2010 [24]	310 prefrail or frail elderly; ≥65 years	6 months *Intervention (I)*: medication adjustment, exercise instruction, nutrition support, physical rehabilitation, social worker consultation, and specialty referrals *Control (C)*: received screening evaluation only	Change in frailty status acc. to the CHS criteria (Fried et al.) [5]. *I*: Same: 83.7%; Improvement: 7.8%; Decline: 8.5% *C*: Same: 82.9%; Improvement: 6.4%; Decline: 10.7% → no sig. group differences
Tarazona-Santabalbina et al. 2016 [25]	100 sedentary frail elderly people from rural care centers	6 months *Intervention (I)*: 5 × a week (66 min), group training balance (10–15 min), aerobic (initially at 40% of maximum heart rate increasing to 65%), strength training (initially at 25% of 1 repetition maximum to 75%), and stretching *Control (C*): received no training and attended the regular primary care program	Number of frailty criteria acc. to the CHS criteria (Fried et al.) [5]. *I*: 3.6 (SD: 0.8)–1.6 (SD: 0.9) *C*: 3.8 (SD: 0.6)–3.8 (SD: 0.3) Edmonton criteria [20] *I*: 8.7 (SD: 2.5)–7.7 (SD: 2.0) *C*: 8.5 (SD: 2.1)–9.3 (SD: 2.3) → 31.4% (95% CI: 20.3–45.0) of the I reversed frailty; no participant in the C

*Rep* repetitions, *SD* standard deviation, *CI* confidence interval, *CHS* Cardiovascular Health Study, *sig.* significant, *acc.* according

**Table 2:** Overview of the effects of physical activity intervention on muscle strength

Author, year	Sample	Intervention	Main findings
Kwon et al. 2015 [26]	89 prefrail women; ≥70 years	3 months *Training (T)*: 1 × a week; group training warm-up, strength training (1 set, 5–10 rep), balance training (20–45 min), and cool-down (5–10 min) *Training and nutrition (TN)*: training & cooking classes (2–3 h) once a week *Control (C)*: 3 sessions of health education	Change in mean handgrip strength (kg) *T*: +2.3 (SD: 3.1) *TN*: +1.2 (SD: 4.0) *C*: +0.4 (SD: 2.6) → sig. increase in the T group
Gine-Garriga et al. 2010 [27]	41 frail individuals; 80–90 years	3 months *Functional circuit training (FCT)*: 2 × a week (45 min), group training warm-up, walking at usual pace (10 min), cool-down, stretching (5 min) *Control (C)*: continue their routine daily activities and usual care from their primary-care practice	Mean maximal isometric quadriceps and hamstring strength (Nm/kg) *FCT*: Baseline: 0.8 (SD: 0.1); 12 weeks: 0.9 (SD: 0.1); 36 weeks: 0.8 (SD: 0.1) *C*: Baseline: 0.8 (SD: 0.1); 12 weeks: 0.7 (SD: 0.1); 36 weeks: 0.6 (SD: 0.1) → sig. group and time effect
Tieland et al. 2012 [28]	62 frail individuals; ≥65 years	3 months *Strength training and nutrition supplementation (TS)*: 2 × a week; individual warm-up (5 min, cycle ergometer), strength training (4 sets on the leg-press and the leg-extension, 3 sets on the chest press, lat pulldown, pec deck and vertical row machine; 50% of the RM increased to 75%) and 2 × daily protein supplementation *Control (C)*: no training and placebo supplementation	Mean handgrip strength (kg) *TS*: 25.9 (95% CI: 22.3; 29.5)–27.2 (95% CI: 23.6; 30.9) *C*: 26.7 (95% CI: 23.1; 30.3)–26.7 (95% CI: 23.1; 30.3) → no sig. group and time difference Mean leg extensor strength (kg) *TS*: 56.0 (95% CI: 49.5; 62.7)–70.0 (95% CI: 62.7; 77.3) *C*: 58.3 (95% CI: 51.7; 64.9)–74.1 (95% CI: 66.8; 81.4) → no sig. group difference
Vestergaard et al. 2008 [29]	61 frail women; ≥75 years	5 months *Training (T)*: 3 × a week with a video tape warm-up (15 min, focusing on flexibility and dynamic balance exercises), strength training (6 min, using elastic bands), aerobic exercises (5 min) *Control (C)*: no intervention at all	Mean handgrip strength (kg) *T*: 17.5 (SD: 5.5)–20.5 (SD: 5.6) *C*: 19.1 (SD: 4.3)–19.8 (SD: 4.5) → no sig. group difference Mean biceps brachii strength (kg) *T*: 6.7 (SD: 2.0)–7.4 (SD: 2.4) *C*: 6.8 (SD: 1.7)–6.9 (SD: 1.7) → no sig. group difference
Haider et al. 2017 [30]	80 prefrail or frail persons; ≥65 years	3 months *Physical training and nutritional (PTN)*: 2 × a week; home visits by trained lay volunteers strength exercises (6 exercises, 2 sets, 15 rep until muscular exhaustion) and nutritional support *Social support (SoSu)*: 2 × a week; home visits with social support	Change in mean handgrip strength (kg) *PTN*: +2.4 (95% CI: 1.0–3.8) *SoSu*: +0.8 (95% CI: −0.4–2.0) → sig. increase in the PTN group; no sig. group difference
Chin et al. 2001 [31]	157 frail elderly persons; ≥70 years	17 weeks *Exercise (E)*: 2 × a week (45 min); group training Strength training (increasing intensity 6–8 on a 10-point perceived exertion scale) *Nutrition (N)*: several fruit and dairy products were enriched with vitamins and minerals; the E and C group received the same products, but the foods were not enriched *Both (B)*: Exercise and nutritional intervention *Control (C)*: No intervention	Change in mean handgrip strength (kgf) *Exercise (E or B)*: 0 (10th–90th percentile: −3 to 5) *No Exercise (N or C)*: +1 (10th–90th percentile: −3 to 4) *N* *+* *B*: +1 (10th–90th percentile: −3 to 5) *E* *+* *C*: 0 (10th–90th percentile: −3 to 4) → no sig. differences between exercises vs. no exercise Change in quadriceps strength (kgf) *Exercise (E or B)*: +1.5 (10th–90th percentile: −4.9 to 8.7) *No Exercise (N or C)*: +0.3 (10th–90th percentile: −4.6 to 6.0) *N* *+* *B*: +1.3 (10th–90th percentile: −3.8 to 7.8) *E* *+* *C*: +0.9 (10th–90th percentile: −5.4 to 6.8) → no sig. differences between exercises vs. no exercise
Ng et al. 2015 [32]	246 prefrail and frail individuals; ≥65 years	6 months *Nutrition supplementation (S)*: 1 × a day hypercaloric supplement *Training (T)*: 2 × a week (90 min) strength (8–10 muscle groups, 8–15 rep, starting with <50% of the RM increasing to 80%) and balance training; after 12 weeks a home-based program was conducted *Training & supplementation (TS)*: combination of both *Control (C)*: standard care from health & aged care services + sweetened, vanilla-flavored liquid, 2 capsules & 1 tablet identical in appearance to the active supplements	Change in mean knee extension strength (kg) *S*: +0.97 (95% CI: 0.15; 2.09) *T*: +2.75 (95% CI: 1.66; 3.83) *TS*: +2.67 (95% CI: 1.58; 3.76) *C*: +0.02 (95% CI: −1.08; 1.12) → sig. group difference between T and C; and TS and C
Chandler et al. 1998 [33]	100 frail people; ≥65 years	10 weeks *Intervention (I)*: 3 × a week, in-home program strength training (progressive, lower extremity using dynaband and body weight) *Control (C)*: no intervention	Change in mean right knee extension strength (Nm) *I*: +4.9 (SD: 14); *C*: −0.7 (SD: 8.2) Change in mean right knee flexion strength (Nm) *I*: +4.6 (SD: 7.1); *C*: +0.3 (SD: 4.8) Change in mean right dorsiflexion strength (Nm) *I*: +0.8 (SD: 3.1); *C*: −0.3 (SD: 2.1) Change in mean right plantar flexion strength (Nm) *I*: +3.1 (SD: 6.4); *C*: −0.3 (SD: 5.7) → *I*: 9% to 16% strength gain; *C*: 1% gain to 3% decline

*Rep* repetitions, *SD* standard deviation, *CI* confidence interval, *sig.* significant, *kgf* kilogramforce

**Table 3:** Overview of the effects of physical activity intervention on physical performance

Author, year	Sample	Intervention	Main findings
Kwon et al. 2015 [26]	89 prefrail women; ≥70 years	3 months *Training (T)*: 1 × a week; group training warm-up, strength training (1 set, 5 progressing to 10 rep.), balance training (20–45 min), cool down (5–10 min) *Training & nutrition (TN)*: 1 × a week (2–3 h), training & cooking classes *Control (C)*: 3 sessions of health education	Change in stork stand (s) *T*: −2.0 (SD: 16.9); *TN*: +2.9 (SD: 18.6); *C*: −0.4 (SD: 11.9) → no sig. changes in any group **Change in usual walking speed (m/s)** *T*: +0.1 (SD: 0.6); *TN*: +0.2 (SD: 0.3); *C*: +0.1 (0.4) → no sig. change in any group
Tieland et al. 2012 [28]	62 frail individuals; ≥65 years	3 months *Strength training & nutrition supplementation (TS)*: 2 × a week, individual warm-up (5 min, cycle ergometer), strength training (4 sets on the leg-press and the leg-extension, 3 sets on the chest press, lat pulldown, pecdeck and vertical row machine; 50% of the RM increased to 75%) & 2 × daily protein supplementation *Control (C)*: no training & placebo supplementation	Mean chair rise (sec) *TS*: 15.6 (95% CI: 13.0; 18.1)–13.6 (95% CI: 10.9; 16.3) *C*: 17.3 (95% CI: 14.8; 19.9)–16.4 (95% CI: 13.9; 19.0) → no sig. group difference Mean points Short Physical Performance Battery *TS*: 8.0 (95% CI: 7.2; 8.9)–9.2 (95% CI: 8.3; 10.1) *C*: 7.9 (95% CI: 7.0; 8.8)–8.3 (95% CI: 7.3; 9.1) → no sig. group difference
Ng et al. 2015 [32]	246 prefrail and frail individuals; ≥65 years	6 months *Nutrition supplementation (S)*: 1 × a day hypercaloric supplement *Training (T)*: 2 × a week (90 min) strength (8–10 muscle groups, 8–15 rep starting with <50% of the RM increasing to 80%) and balance training; after 12 weeks a home-based program was conducted *Training & nutrition supplementation (TS)*: combination of both *Control (C)*: standard care from health & aged care services + sweetened, vanilla-flavoured liquid, 2 capsules & 1 tablet identical in appearance to the active supplements	**Mean change in gait speed (s)** *S*: −0.8 (95% CI: −1.2; −0.4) *T*: −1.1 (95% CI: −1.5, −0.7) *TS*: −0.5 (95% CI: −1.0, −0.1) *C*: −0.7 (95% CI: −1.1; −0.3) → sig. group difference between the T and the C group
Vestergaard et al. 2008 [29]	61 frail women; ≥75 years	5 months *Training (T*): 3 × a week; with a video tape warm-up (15 min, focusing on flexibility and dynamic balance exercises), strength training (6 min, using elastic bands), aerobic exercises (5 min) *Control (C)*: no intervention at all	**Mean semi balance (s)** *T*: 11.9 (SD: 8.0) to 15.5 (SD: 7.3) *C*: 13.2 (SD: 7.5) to 13.3 (SD: 8.1) → no sig. group difference **Mean chair rise (s)** *T*: 19.3 (SD: 11.6) to 14.1 (SD: 8.5) *C*: 16.4 (SD: 5.3) to 16.3 (SD: 6.2) → no sig. group difference **Mean physical performance test (score)** *T*: 16.3 (SD: 5.6) to 18.1 (SD: 5.8) *C*: 17.0 (SD: 4.9) to 17.4 (SD: 5.5) → no sig. group difference
Haider et al. 2017 [30]	80 prefrail or frail persons; ≥65 years	3 months *Physical training and nutritional (PTN)*: 2 × a week; home visits by trained lay volunteers strength exercises (6 exercises, 2 sets, 15 rep until muscular exhaustion) and nutritional support *Social support (SoSu)*: 2 × a week; home visits with social support	Change in mean points of the Short Physical Performance Battery *PTN*: +1.2 (95% CI: 0.3–2.1) *SoSu*: +0.5 (95% CI: 0.1–0.9) → sig. improvements in both groups; sig. group differences
Cameron et al. 2013 [22]	216 frail adults; ≥70 years	12 months *Intervention (I)*: multifactorial, individually tailored treatment 1) If weight loss criterion was met, a dietician came. 2) If exhaustion criterion was met and the Geriatric Depression Scale score was high, a psychiatrist or psychologist came. 3) If weakness, slowness or low energy expenditure criteria was present, 10 home-based physiotherapy sessions were performed *Control (C)*: usual care, including health and aged care services	Mean points of the Short Physical Performance Battery *I*: Baseline: 5.2 (SD: 1.9); 3 months: 5.4 (SD: 2.3); 12 months: 5.8 (SD: 2.8) *C*: Baseline: 5.7 (SD: 2.1); 3 months: 5.7 (SD: 2.3); 12 months: 4.7 (SD: 2.9) → sig differences between I and C group after 12 months
Zech et al. 2012 [34]	69 prefrail adults; 65–94 years	3 months *Strength training (T)*: 2 × a week; individual 9 strength exercises (2 sets, 2 min rest, intensity was increased continuously) *Muscle power training (PT)*: same exercises as described above; the concentric phase was conducted rapidly, the eccentric phase as slowly *Control (C)*: no intervention	Mean points of the Short Physical Performance Battery *T*: 8.8 (SD: 2.4) to 9.7 (SD: 2.2) *PT*: 9.0 (SD: 2.1) to 10.1 (SD: 2.3) *C*: 10.2 (SD: 2.1) to 9.7 (SD: 2.1) → sig. difference in the T and the PT group
Chin et al. 2001 [31]	157 frail elderly people; ≥70 years	17 weeks *Exercise (E)*: 2 × a week (45 min), group exercise Strength training (increasing intensity 6–8 on a 10-point perceived exertion scale) *Nutrition (N)*: several fruit and dairy products were enriched with vitamins and minerals; the E and C group received the same products, but the foods were not enriched *Both (B)*: Exercise and nutritional intervention *Control (C)*: No intervention	Change in mean chair stands (rep) *E* *+* *B*: −2.3 (10th–90th: −7.7 to 1.4) *N* *+* *D*: −1.0 (10th–90th: −6.4 to 3.8) *N* *+* *B*: −1.8 (10th–90th: −7.8 to 2.2) *E* *+* *C*: −1.9 (10th–90th: −6.0 to 2.5) → sig. differences between E + B and *N* + D group Change in mean walking speed (m/s) *E* *+* *B*: +0.6 (10th–90th: 0.1) *N* *+* *D*: 0.0 (10th–90th: 0.4) *N* *+* *B*: 0.0 (10th–90th: 0.1) *E* *+* *C*: +0.1 (10th–90th: 0.1) → sig. differences between E + B and *N* + D group **Change in mean tandem stand (s)** *E* *+* *B*: +0.9 (10th–90th: 2.8) *N* *+* *D*: −0.8 (10th–90th: 3.4) *N* *+* *B*: +0.3 (10th–90th: 3.3) *E* *+* *C*: −0.1 (10th–90th: 3.1) → sig. differences between E + B and *N* + D group
Gine-Garriga et al. 2010 [27]	41 frail individuals; 80–90 years	3 months *Functional circuit training (FCT)*: 2 × a week (45 min), group training warm-up, walking at usual pace (10 min), cool-down, stretching (5 min) *Control (C)*: continue their routine daily activities and usual care from their primary-care practice	**Mean stand-up Test (s)** *FCT*: Baseline: 19.6 (SD: 0.7); 12 weeks: 15.6 (SD: 0.7); 36 weeks: 17.8 (SD: 0.7) *C*: Baseline: 17.1 (SD: 0.9); 12 weeks: 17.9 (SD: 0.9); 36 weeks: 17.5 (SD: 1.1) → sig. group and time effect **Mean modified timed up-and-go (s)** *FCT*: Baseline: 38.0 (SD: 1.3); 12 weeks: 35.0 (SD: 1.3); 36 weeks: 37.5 (SD: 1.3) *C*: Baseline: 39.3 (SD: 1.4); 12 weeks: 41.3 (SD: 1.4); 36 weeks: 42.0 (SD: 1.4) → sig. group and time effect
Tarazona-Santabalbina et al. 2016 [25]	100 sedentary frail elderly from rural care centers	24 weeks *Intervention (I)*: 5 × a week (66 min); group exercise balance (10–15 min), aerobic (initially at 40% of maximum heart rate increasing to 65%), strength training (initially at 25% of 1 repetition maximum to 75%), and stretching *Control (C*): received no training and they attended the regular primary care program	**Mean Tinetti (s)** *I*: 23.5 (SD: 4.4) to 24.5 (SD: 4.4) *C*: 24.7 (SD: 3.4) to 21.7 (SD: 4.5) **Mean point Short Physical Performance Battery** *I*: 8.6 (SD: 2.0) to 9.5 (SD: 1.8) *C*: 8.6 (SD: 1.7) to 7.1 (SD: 2.8) → sig. improvements in the I, deteriorations in the C
Chan et al. 2012 [23]	117 prefrail or frail adults; 65–79 years	3 months *Exercise and nutrition (EN)*: 3 × a week (1 h); structured exercise course warm up (15 min), brisk walks (10 min), stretching, strength training (10–15 rep., rubber band and bottled water), balance training, cool down (5 min) *Problem solving therapy (PST)*: 6 session of evidence-based physiotherapy	**Change in mean leg stand (s)** *EN*: 3 months: +2.9 (SD: 9.2); 6 months: +2.6 (SD: 8.4); 12 months: +3.7 (SD: 9.2) *PST*: 3 months: +2.4 (SD: 8.9); 6 months: +3.1 (SD: 8.9); 12 months: +4.3 (SD: 10.2) → sig. increase in both groups

*Rep* repetitions, *SD* standard deviation, *CI* confidence interval, *RM* repetition maximum

**Table 4:** Overview of the effects of physical activity intervention on muscle mass

Author, year	Sample	Intervention	Main findings
Tieland et al. 2012 [28]	62 frail individuals; ≥65 years	3 months *Strength training & nutrition supplementation (TS)*: 2 × a week, individual warm-up (5 min, cycle ergometer), strength training (4 sets on the leg-press and the leg-extension, 3 sets on the chest press, lat pulldown, pec-deck and vertical row maschin; 50% of the RM increased to 75%) and 2 × daily protein supplementation *Control (C)*: no training and placebo supplementation	Mean lean mass (kg) *TS*: 47.2 (95% CI: 43.5; 50.9)–48.5 (95% CI: 44.8; 52.1) *C*: 45.7 (95% CI: 42.1; 49.2)–45.6 (95% CI: 42.1; 49.2) → sig. group and time interaction Mean appendicular lean mass (kg) *TS*: 20.1 (95% CI: 18.3; 21.8)–20.4 (95% CI: 18.6; 22.1) *C*: 19.3 (95% CI: 17.6; 20.9)–19.3 (95% CI: 19.7; 21.0) → sig. group and time interaction
Zech et al. 2012 [34]	69 prefrail older adults; 65–94 years	3 months *Strength training (T)*: 2 times a week; individual 9 strength exercises (2 sets, 2 min rest, intensity was increased continuously) *Muscle power training (PT)*: same exercises as described above; the concentric phase was conducted rapidly, the eccentric phase as slowly *Control (C)*: no intervention	Mean appendicular lean mass (kg) *T*: 17.9 (SD: 3.3)–18.0 (SD: 3.3) *PT*: 19.2 (SD: 4.4)–19.1 (SD: 4.2) *C*: 17.1 (SD: 2.6)–17.5 (SD: 2.6) → no sig. change in any group
Chan et al. 2012 [23]	117 prefrail or frail adults; 65–79 years	3 months *Exercise and nutrition (EN)*: 3 × a week (1 h); group training warm up (15 min), brisk walks (10 min), stretching, strength training (10–15 rep., rubber band and bottled water), balance training, cool down (5 min) *Problem solving therapy (PST)*: 6 session of evidence-based physiotherapy	Change in mean fat free mass (kg) *EN*: 12 months: −0.5 (SD: 1.4) *PST*: 12 months: −0.6 (SD: 1.3) → sig. decrease in all groups
Haider et al. 2017 [30]	80 prefrail or frail persons; ≥65 years	3 months *Physical training and nutritional (PTN)*: 2 × a week; home visits by trained lay volunteers strength exercises (6 exercises, 2 sets, 15 rep until muscular exhaustion) and nutritional support *Social support (SoSu)*: 2 × a week; home visits with social support	Change appendicular skeletal muscle mass (kg) *PTN*: +0.3 (95% CI: −0.2; 0.7) *SoSu*: +0.2 (95% CI: −0.1; 0.5) → no sig. change in any group

*Rep* repetitions, *SD* standard deviation, *CI* confidence interval

### Does PA reduce frailty in community-dwelling prefrail and frail persons?

The main characteristics of studies exploring the effects of PA interventions on frailty status are presented in Table 1. In total, five studies investigated whether PA may have beneficial effects on the frailty status [21–25]. Overall, 4 studies used the CHS criteria [22–25], with other 2 using SHARE Frailty Index (SHARE-FI) [21] and Edmonton criteria [25].

Studies varied in training protocols, with all studies providing standardized interventions. There was a variation in the provided frequency for PA with a range of 2–5 times per week between studies. All studies included strength training in their interventions, with two studies additionally including aerobic training [23, 25]. In terms of the control group interventions, two studies had an active control group (meaning that participants in the control group did receive some intervention in terms of cognitive training or physiotherapy), while the rest did not provide any additional support to the controls. A study conducted by Cameron et al. [22] was the only one to provide an individually tailored approach, where the intervention was modified based on the present frailty criteria including an interdisciplinary team. Most studies provided both PA and nutritional support interventions, with only one study not providing nutritional support [25]. Finally, only one study reported an intervention that was supplied by non-professional trained volunteers [21]. The effect size reported in that study was similar to other studies reporting significant changes and differences between groups.

### Does PA improve muscle strength in community-dwelling prefrail and frail persons?

Of the identified studies eight focused on muscle strength as the outcome of interest [26–33]. A detailed overview is given in Table 2. Of the studies five provided both PA and nutritional support interventions [26, 28, 30–32], with a study by Kwon et al. [26] having distinguished groups (one receiving only PA and one both PA and nutritional support). Several of the included studies included nutritional supplementation in view of vitamins and minerals and protein supplementation [28, 31, 32]. A study by Tieland et al. [28] provided an intervention comprising strength training twice per week (aimed at chest, back and legs) with twice daily protein supplementation and the control group received no training or placebo supplementation. The study reported an increase in both mean handgrip strength and mean leg extensor strength but found no significant differences between groups. Half of the identified studies reported no significant differences between the groups [28–31], irrespective of follow-up duration or nutritional support.

There were two studies that only had female participants [26, 29]. Both studies found an increase in mean handgrip strength; however, Vestergaard et al. [29] reported no significant differences between the groups while Kwon et al. [26] reported a significant increase in handgrip strength only in PA group, not in the group that received PA and nutritional support.

### Does PA improve physical performance in community-dwelling prefrail and frail persons?

With a total of 11 studies describing on some aspects of physical performance, this outcome was the most reported one. Most studies reported significant changes between groups and over time [22, 23, 25, 27, 30–32, 34]. Interestingly, from the three studies reporting no change in physical activity performance, two were performed with female participants only, barring an unknown bias or gender specific factors. There are also discrepancies in the ways that physical performance was measured. While six studies included the Short Physical Performance Battery (SPPB), others included less comprehensive tests, such as a change in usual walking speed, chair stand test or the tandem stand test. These differences between the tests could also prohibit direct comparisons of the results. Furthermore, differences in reported interventions were found between studies; as some studies included both nutritional and PA aspects. In that respect there seems to be a discrepancy as both studies with nutritional and PA interventions, as well as those that only had PA interventions, reported significant and non-significant outcomes. Overall there seems to be evidence pointing to a positive role of PA on physical performance, however, less convincing than in the reduction of frailty outcome (Table 1).

### Does PA increase muscle mass in community-dwelling prefrail and frail persons?

A total of four studies included changes in muscle mass as outcome [23, 28, 30, 34]. All these studies focused on strength training and nutritional support or supplementation, with one study including a special intervention group for muscle power [34]. The study by Tieland et al. [28] was also the only study that provided protein supplementation. All studies had a follow-up period of 3 months but only the study by Tieland et al. [28] reported significant over time and between group results, and the study by Chan et al. [23] reported a decrease in fat-free mass in both groups during the follow-up period. Therefore, based on the results of the included studies there seems to be no evidence to support the hypothesis of PA increasing muscle mass in prefrail and frail community dwelling persons.

## Discussion

The results of this narrative review indicate that PA interventions in frail older community dwelling adults are effective for some but not all of the investigated outcomes. Most agreement between the studies is on the reduction of frailty and physical performance outcomes, where the majority of identified studies report favorable effects of PA (Table 1 and 3). In the muscle strength outcome there is limited evidence of the effectiveness of PA interventions, while there seems to be no evidence to support the increase in muscle mass in frail or prefrail older people through PA interventions.

The inhomogeneity of the results might be due to the differences in training protocols (method, intensity, duration, frequency, follow-up); some interventions used a multifactorial training, others have conducted strength training only. According to the literature strength training may be of most importance, due to associations of frailty and sarcopenia [11]. As can be taken from Tables 1–4; in most studies a multicomponent training was performed. These results are in line with the review of Theou et al. [19], showing that interventions comprising a 3-month long multicomponent training, 3 times per week for 1 h sessions, were the most commonly ones used. It was conspicuous that in the included studies the intensity, which is one of the cornerstones of the effectiveness of an intervention, greatly differs or is not described in detail.

In the presented studies different training frequencies and follow-up times, ranging from 10 weeks to 12 months (Tables 1–4) were also used. Furthermore, there were disparities in the included study participants; the age of recruited participants differed, and in some studies only females were included. Another methodological issue that prohibits direct comparisons of the studies is in the varying ways that frailty was defined in the included studies; the majority of the studies used the CHS criteria [5], but there are also studies assessing frailty with the SHARE-FI [21, 30], or the Edmonton criteria [25].

When looking at the included studies, there were also differences in the intervention delivery; some studies performed a group training, others conducted an individually tailored program. Furthermore, some studies provided comprehensive interventions delivered in health care institutions by a variety of professionals, while some provided at home interventions by lay volunteers, some provided videos of the exercises without supervision. Authors could not identify any differences in the effects of the interventions, caused by the intervention delivery; however, according to Apostolo et al. [18] physical exercise programs were only effective in reducing frailty, when conducted in groups and there is a lack of efficacy when delivered one to one. Differences in results might be that Apostolo et al. included old people and not only frail individuals. There are also differences in the people who delivered the intervention. In one study the intervention was conducted by trained lay volunteers [21, 30]. In all other trials the intervention was performed by professionals.

The fact that some studies provides only limited information on the intervention program makes it difficult to compare or comment on possible reasons for inhomogeneity. For example, the study by Li et al. [24], reported trends that were not significant for the intervention group in reduction of frailty. Additionally, the majority of the trials added nutrition interventions (focused on either supplementation or comprehensive nutritional changes), making it difficult to distinguish the real effects of the PA intervention.

Finally, it ought to be mentioned that various examinations used different tools to measure the effectiveness of the PA intervention; some assessed changes in physical performance using SPPB, whereas others have conducted the chair stand test or the tandem stand test (Table 3). For measuring muscle strength most authors used handgrip strength, others measured lower extremity strength by assessing changes in the knee extension or the quadriceps (Table 2).

An interesting finding was that physical activity was effective in gaining muscle strength, and the studies are relative consistent in this respect; however, they failed to increase muscle mass. A possible reason for the non-response of muscle mass is that in prefrail and frail older adults only muscle quality, muscle function can increase with no effect on muscle mass [35, 36]. Another reason could be that although a high intensity (>60% of the maximum strength) is usually intended, in the authors’ experience participants tend to perform the training with a lower intensity.

When interpreting the results, it has to be considered that these results only apply for community-dwelling prefrail or frail persons. According to the literature community-dwelling older adults always report better results than those residing in institutions [19]. This may be due to a higher degree of independency but also the drive and motivation for PA intervention compliance might be higher.

The major strength of this narrative review is the chosen inclusion criteria; only studies of frail or prefrail community-dwelling people, as well as only RCTs providing one of the highest levels of evidence, were included. The major limitations of the review are also related to the included papers, which due to a variety of measurements used makes it hard to make clear comparisons. To make the results of this narrative review more meaningful further systematic reviews should be done.

## Conclusion

Although there are no decisive results showing the impact of PA interventions on the outcomes reported here, most of the identified studies demonstrated a positive effect especially in reduction of frailty, improvements in physical performance and to a lesser degree increase in muscle strength. There seems to be, however, no evidence to support the increase in muscle mass in frail or prefrail older people through PA interventions. The fact that some interventions are effective while others are not, might be due to the differences in the study protocols (training protocol, intensity, frequency, delivering method, follow-up time, measuring tools). Future studies need to focus on explaining the positive effects of PA on frailty status and frailty-related outcomes separately and identify the underlying mechanisms.
